# Long-term sequelae of COVID-19 (myalgic encephalomyelitis): An international cross-sectional study

**DOI:** 10.1097/MD.0000000000031819

**Published:** 2022-11-11

**Authors:** Nour Shaheen, Ahmed Shaheen

**Affiliations:** a Department of Medicine, Alexandria University, Alexandria, Egypt.

**Keywords:** chronic fatigue syndrome, long COVID-19 syndrome, myalgic encephalomyelitis, symptoms of COVID-19

## Abstract

As a result of prolonged effects on multiple organs, recovery from COVID-19 caused by SARS-CoV-2 cannot be verified. This study seeks to understand chronic and acute long-term symptoms of COVID-19 lasting from a few weeks after diagnosis. The study also aims to gain insight into prevalence of chronic fatigue syndrome/myalgic encephalomyelitis, a potentially comorbid condition for several months after the infection, in addition to taking a broad perspective on rare symptoms that may have developed during or after the infection.

**Study design:**

Cross-sectional questionnaire\descriptive study.

**Methods:**

The questionnaire was developed to assess the long-term effects of the global pandemic of COVID-19 using DePaul Symptom Questionnaire-2. The DePaul Symptom Questionnaire, Patient Health Questionnaire, and other symptoms that have been introduced by literature review.

**Discussion:**

A large cohort of people from all over the world will be examined to understand the differential effects of people who have experienced COVID-19, as well as the potential occurrence of ME. In total, 20,000 COVID patients are expected to be included in the study by Sep 1, 2022. Patients who have experienced COVID-19 will be asked about their persistent symptoms from 1 week up to more than 6 months after catching or recovery from the infection.

## 1. Introduction

After the outbreak of COVID-19 caused by SARS-CoV-2 in Wuhan, China, the pandemic has spread all over the world.^[1]^ The COVID-19 impacted the lives and health of millions of people worldwide, causing 3.97 million deaths and more than 183 million confirmed cases. After recovery, the disease of COVID-19 may leave persistent symptoms.^[2]^ These symptoms last for some time, even after the recovery. Even some people who had mild symptoms during COVID-19 continue to have long-term effects after initial recovery. These people are called “long haulers,” and these symptoms are called long COVID-19.^[3]^ Despite nucleic acid tests being negative, long haulers still suffer from permanent symptoms due to multiorgan dysfunction.^[3]^ A prolonged COVID-19 infection affects multiple organs, including respiratory, cardiovascular, neurological, gastrointestinal, and musculoskeletal systems.^[3]^ There are only a few studies that have examined the symptoms and experiences of COVID-19 patients. According to The Centers for Disease Control and Prevention (CDC), after 3 weeks one-half of the 300 PCR-positive SARS-CoV-2 patients had permanent symptoms.^[3]^ Symptoms of long-COVID include shortness of breath, cough, myalgias, disturbances in taste and smell, fatigue, fever, chills, and, less commonly, rhinitis, gastrointestinal symptoms, cognitive impairment, sleep disturbances, symptoms of post-traumatic stress disorder, muscle pain, concentration difficulties, and headaches. Additionally, some cases reported by Canadians described unusual symptoms, including a Beau line rash.^[4]^

In 1 out of 10 patients with the infection, the symptoms usually persist and last for 3–4 weeks of acute symptoms, but they may last for more than 12 weeks after the infection.^[5,6]^ According to Dr Anthony Fauci,“ patients with COVID-19 can develop a post-viral syndrome that’s very strikingly similar to Myalgic encephalomyelitis/chronic fatigue syndrome.”^[5,6]^ In case the fatigue persists for 6 months, it is called myalgic encephalomyelitis/chronic fatigue syndrome (ME/CFS). Although 6 months is no longer required for ME diagnosis according to 2011’s ME international Consensus Criteria, it is still common in literature.^[7]^

Recently, there has been global concern about the effectiveness and safety of COVID-19 vaccines. According to WHO, the most common side effects reported about COVID-19 vaccines were fever, fatigue, headache, muscle pain, chills, diarrhea, and pain at the injection site. These side effects are more or less like some post-COVID-19 infection symptoms, so we found it logical to add a section to gather information about the vaccine status, type, and timing with the symptoms.^[8]^

## 2. The study

### 2.1. Aims

#### 2.1.1. Primaryoutcome measures.

Assessing and understanding acute and chronic long COVID-19 symptoms based on a questionnaire asking different questions on testing, diagnosis, and treatments. [Time Frame: once through study completion, an average of 1 year].

List of relevant morbidities, testing, diagnosis, and treatments assessed as present/not present by medical interview (e.g., respiratory, cardiac, neurological, psychiatric diseases by interview and medical records).

#### 2.1.2. Secondary outcome measures.

Assessing the long-term sequelae effects, depression other comorbidities following COVID-19 vaccination. [Time Frame: once through study completion, an average of 1 year].

### 2.2. Methods and analysis

The study will screen patients’ backgrounds, testing, symptoms, and long-term symptoms based on validated scaled studies that assess ME and depression by DePaul Symptom Questionnaire (DSQ)-2 questionnaire and Patient Health Questionnaire-9 and other symptoms that have been introduced by medical interviews.^[9]^

#### 2.2.1. Study design: cross-sectional study.

##### 2.2.2.1. Time.

September 2021 to September 2022.

##### 2.2.2.2. Study settings.

This large-scale, international cross-sectional study is being conducted by ICRAA Team with collaboration with 200 researchers in 28 countries (India, Egypt, Pakistan, Brazil, Nigeria, Bangladesh, Algeria, UK, Perú, Lithuania, UAE, Jordan, Afghanistan, Yemen, Tunisia, Sudan, Turkey, Syria, Iraq, Kosovo, Morocco, Bahrain, Ecuador, Iran, United States, Palestine, Japan) around the world (Table 1).

**Table 1:** List of participating countries in the long COVID study.

Africa	America	Asia	Europe
Algeria	Brazil	Bangladesh	UK
Egypt	Ecuador	Syria	Lithuania
South Africa	Peru	India	Kosovo
Tunisia	United States	Iran	Turkey
Nigeria		Iraq	
Morocco		Bahrain	
		Japan	
		Palestine	
		Pakistan	
		Yemen	
		Afghanistan	
		UAE	
		Jordan	

The study will be conducted in centers or hospitals worldwide after getting the Institutional Review Board worldwide by the lead local investigator.

##### 2.2.2.3. Study population.

Persons who have had a confirmed (diagnostic/antibody positive) test for COVID-19 infection (still suffering or suffering symptoms) for more than 1 week up to more than 6 months of 18 years of age or older.

##### 2.2.2.4. Sample size calculation

In order to ensure that all analyses planned to be conducted without any power issues can be carried out, we selected a sample size of 20,000 participants. The sample size for India, for example, will be large. Person-centered analyses and large-scale analyses will be done, but power calculations and priori sample size calculations will be applied to refine the field.^[10–12]^ Accordingly, there will not be a limit on the number of hospitals or centers participating in the study.

##### 2.2.2.5. Study instrument and questionnaire design process

Demographics and baseline characteristics.
DSQ-2: The DSQ was designed to assess the symptomatology and case definition of individuals with ME and CFS).^[9]^ It will be used to assess ME/CFS symptoms such as fatigue, post-exercise malaise, sleep, pain, neurological/cognitive impairments, as well as autonomic, neuroendocrine, and immune symptoms using a short version of the DSQ that consists of 14 questions. Each item has 5-point Likert-type scales to be rated by the participant. Frequency scores are associated with the following descriptors: 0 = none of the time, 1 = a little of the time, 2 = about half of the time, 3 = most of the time, and 4 = all the time. Severity scores are defined as: 0 = symptom not present, 1 = mild, 2 = moderate, 3 = severe, 4 = very severe.^[13]^
Patient Health Questionnaire consists of 9 questions about feeling in the past 2 weeks. Answers are on a 4-rate scale, ranging from not^[13]^at all to nearly every day. Each answer has a score then the total score is calculated. A score of 1–4 indicates minimal depression. A score of 5–9 indicates mild depression. A score of 10–14 indicates moderate depression. A score of 15–19 shows moderately severe depression. A score of 20–27 indicates severe depression.^[14]^
Other symptoms have been introduced by a literature review.^[5,6,15]^

##### 2.2.2.6. Measures.

The study questionnaire consists of 10 sections with 169 items (Table 2) provided in (Supplementary material 1, Supplemental Digital Content, http://links.lww.com/MD/H979).

**Table 2:** Complete list of variables in the long COVID-19 questionnaire in order of presentation for participants.

Variable (Scale, Abbreviation)	Number of items
Demographics and baseline characteristics	15
COVID-19 testing	4
COVID-19 experience	6
Hospitalization	3
Treatments	2
Vaccination	5
DePaul Symptom Questionnaire—short form (DSQ—SF)	14
(SF-36) Survey to measure substantial reduction requirement in the case definitions	36
Patient Health Questionnaire (PHQ-9)	9
Other long COVID-19 symptoms	57

PHQ-9 = Patient Health Questionnaire.

##### 2.2.2.7. Study conduct.

Teams from local health departments will collect data on confirmed cases of COVID-19. Multiple groups from the same center or institution will be recruited, and new collaborators can join while conducting the study. Centres dealing with large numbers of confirmed COVID-19 cases can ask for more than a 1-month data collection period. Data will be collected from volunteered COVID-19 patients, depending on a direct physician–patient interview.

Collaboration with the collaborator will involve patients being interviewed at outpatient clinics or by telephone. Data will be collected by local data collectors choosing the most convenient way to reach the patients. Data will be validated twice by local and national leaders. Data collected from the patients should not include any identifiable data that can lead to the patient. Data collectors should be mentored closely by the local hospital leader.

##### 2.2.2.8. Data management.

Data will be submitted to the central team’s secure platform via a Google Forms by the local and national leaders. After that, the collected data will be saved in a safe platform for further analysis.

##### 2.2.2.9. Data analysis.

The study data will be gathered anonymously, and the data will be saved as an excel sheet. Descriptive analysis mode, the frequency for categorical data, and the mean interquartile range will be done. Shapiro–Wilk test will be done for normality. A *t* test will be done to compare between groups, ANOVA for continuous, and Chi-square for the noncategorical variable if the data is normally distributed. If not, Kruskal–Wallis and Mann–Whitney *U* test will be done. Correlation and regression analysis will be considered to look further at the relations between variables.

##### 2.2.2.10. Ethical aspects.

This study will be conducted by the principals of the declaration of Helsinki that guarantee the rights of participants and patients.^[16]^ Furthermore, ethical approval was received from the Ethics committee of NewGiza university in November 2021 (Approval No. N-14-2021) in Egypt, KRL Hospital Islamabad in Pakistan, University of Tobruk (UOT) in Libya, University of Aleppo in Syria and Hospital Riyadh Al-Jariri for fevers and treatment center for corona cases in Yemen.

Also, prior to collecting data about other international settings, the national leader must receive ethical approval from appropriate federal and local governments, hospitals, and centers’ academic bodies. In order to obtain Institutional Review Board approval, the approval process must follow all national and local standards. Government and academic bodies participating in the project will be briefed on the results and findings.

##### 2.2.2.11. Authorship.

The publications resulting from this study will be published under the name Global long COVID Collaborative as one author, encompassing the efforts of all collaborators. All collaborators’ names will be listed, along with their contributions, at the end of the publication. This collaborative authorship model aims to reduce conflicts and encourage collaboration by using a single group name and keeping track of what everyone has done.

## 3. Discussion

Our study is the first international comprehensive study to investigate the long-term effects of COVID-19 “Long COVID” among large cohorts of people, which will have significant impacts on baseline health.

Among the COVID-19 patients who have recovered from their illness for a few weeks to 6 months, the study will investigate many post-COVID-19 symptoms that have been reported in many cases, the study also will determine if CFS is prevalent; The project will provide an extensive screening for long COVID-19 syndrome in a large, international, multilingual population in more than 28 countries across 5 continents. In addition to providing an overview of the public health responses after COVID-19 and enriching the literature review with detailed information about its symptoms, this will serve as a bottom-up approach to policy. As another goal, we wish to develop evidence-based recommendations measures to influence various aspects of public health after the research is complete. Finally, we encourage researchers to apply the measures used and assess approaches to better understand the long-term effects of the globally widespread COVID-19 pandemic.

## Author contributions

**Conceptualization:** Nour Shaheen, Ahmed Shaheen.

**Data curation:** Nour Shaheen, Ahmed Shaheen.

**Formal analysis:** Nour Shaheen, Ahmed Shaheen.

**Funding acquisition:** Nour Shaheen, Ahmed Shaheen.

**Investigation:** Nour Shaheen, Ahmed Shaheen.

**Methodology:** Nour Shaheen, Ahmed Shaheen.

**Project administration:** Nour Shaheen, Ahmed Shaheen.

**Resources:** Nour Shaheen, Ahmed Shaheen.

**Software:** Nour Shaheen, Ahmed Shaheen.

**Supervision:** Nour Shaheen, Ahmed Shaheen.

**Validation:** Nour Shaheen, Ahmed Shaheen.

**Visualization:** Nour Shaheen, Ahmed Shaheen.

**Writing—original draft:** Nour Shaheen, Ahmed Shaheen.

**Writing—review and editing:** Nour Shaheen, Ahmed Shaheen.

## Supplementary Material


