# Supplementary material for: Long-term sequelae of COVID-19 (myalgic encephalomyelitis): An international cross-sectional study
Source: Medicine (Baltimore). 2022 Nov 11;101(45):e31819. doi: 10.1097/MD.0000000000031819 (PMC9665887; doi:10.1097/MD.0000000000031819)
Supplement: Supplementary file 1 [file medi-101-e31819-s001.pdf]

Contents

Tools 1

Demographics and baseline characteristics 1

COVID-19 Testing 3

COVID-19 experience 3

Hospitalization 5

Treatments 5

Vaccination 7

DePaul Symptom Questionnaire – (54 itemsDSQ – SF) 7

Patient Health Questionnaire (PHQ-9) 20

Other symptoms 21

Tools

1. Demographics and baseline characteristics
2. DSQ-2: The DePaul Symptom Questionnaire (DSQ) was developed to assess the symptomatology and case definition fulfillment of individuals with Myalgic encephalomyelitis (ME) and chronic fatigue syndrome (CFS)
3. Patient Health Questionnaire (PHQ-9): it consists of nine questions about feeling in the past 2 weeks. Answers are on a 4-rate scale, ranging from not at all to nearly every day. Each answer has a score then the total score is calculated. A score of 1–4 indicates minimal depression. A score of 5–9 indicates mild depression. A score of 10–14 indicates moderate depression. A score of 15–19 indicates moderately severe depression. A score of 20–27 indicates severe depression
4. Other symptoms that have been introduced by literature review

Demographics and baseline characteristics

What is your height?	
What is your weight?	
In which country do you currently reside?	
What city do you live in? Please include state if applicable. (i.e. New York, NY)	
What type of area do you live in?	Suburban<br>Urban<br>Rural
What age group do you fall into?	18-29<br>30-39<br>40-49

	50-59<br>60-69<br>70-79<br>80+
Sex	Male<br>Female
If applicable, are you pregnant?	Yes<br>No
If applicable, are you 6 months or less postpartum?	Yes<br>No<br>N/A
If applicable, do you have periods/a menstrual cycle?	Yes<br>No, post-menopausal<br>No, other reason<br>N/A
Which of the following best describes your ancestry? Select all that apply.	Asian, South Asian, southeast Asian (Chinese, Asian Indian, Vietnamese, Filipino...)<br>Black (African American, Jamaican, Nigerian, Haitian...)<br>White (German, Italian, English, Polish, French...)<br>Hispanic, Latino, or Spanish Origin (Mexican, Mexican American, Puerto Rican, Cuban...)<br>Indigenous Peoples (Navajo Nation, Blackfeet Tribe, Mayan, Inupiat...)<br>Pacific Islander (Native Hawaiian, Samoan, Fujian, Chamorro...)<br>Middle Eastern, North African (Lebanese, Iranian, Egyptian, Moroccan...)<br>Prefer not to answer<br>Other
Is your household income from all sources?	Less than $10,000<br>$10,000 to less than $20,000<br>$20,000 to less than $25,000<br>$25,000 to less than $35,000<br>$35,000 to less than $50,000<br>$50,000 to less than $75,000<br>More than $75,000<br>Don't know/Not sure
What is your highest educational level achieved?	Less than high school<br>Some high school<br>High school degree or GED<br>Partial college (at least one year) or specialized training<br>Standard college degree<br>Graduate professional degree including masters and doctorate
Are you a healthcare professional?	Yes<br>No
What is your current work status? Select all that apply.	On disability<br>Student<br>Homemaker

	Retired<br>Unemployed<br>Working part-time<br>Working full-time

COVID-19 Testing

Did you have a COVID-19 infection?	Yes<br>No
Did you consult with a physician(s) for your COVID-19 symptoms?	Alternative Medicine doctor<br>Cardiologist<br>Dermatologist<br>Gastroenterologist<br>Hematologist<br>Hospitalist<br>Immunologist/Allergist<br>Infectious disease specialist<br>My primary care doctor/General practitioner<br>Neurologist/Neuroimmunologist<br>Obstetrician-Gynecologist (OB-GYN)<br>Psychiatrist<br>Pulmonologist<br>Rheumatologist<br>Other<br>I have not seen any physician
What type of test was used to test you for COVID-19?	Nasal swab (PCR test)<br>Spit test (PCR test)<br>Swab or spit RAPID test (results within a couple of hours)<br>Don't know
What date were you tested for COVID-19? If you don't know the exact date, please choose your best approximation.	Specific date

COVID-19 experience

When did your symptoms begin?	Specific date
Are you still experiencing symptoms?	Yes<br>No
Recovered - Total Days<br>How many days total did you experience symptoms?	

Lifestyle & Pre-existing Conditions<br>Did you have any of these pre-existing conditions/diagnoses or did you experience any of the following pre-COVID-19?	Food Allergies<br>Environmental Allergies (dust, mold)<br>Chemical Allergies Seasonal Allergies<br>Allergies of unknown origin<br>Other allergies<br>Insomnia<br>Lucid dreams (dreams where you are aware you are dreaming or have some control over what you dream)<br>Nightmares<br>Vivid dreams<br>Night sweats<br>Sleep apnea<br>Acid Reflux Disease<br>Celiac Disease<br>Crohn's Disease<br>Ulcerative Colitis<br>Irritable Bowel Syndrome (IBS)<br>Other GI issues<br>Asthma<br>Chronic Obstructive Pulmonary Disease (COPD)<br>Tuberculosis<br>Eczema<br>Viral skin conditions (cold sores, herpes, warts, molluscum)<br>Dementia<br>Seizures/epilepsy<br>Migraine<br>Amyotrophic lateral sclerosis<br>Parkinson's disease<br>Multiple Sclerosis<br>Peripheral neuropathy<br>Coronary Heart Disease<br>Heart failure<br>Hypertension (high blood pressure)<br>Hypotension (low blood pressure)<br>History of blood clotting<br>History of strokes<br>High cholesterol/hyperlipidemia<br>Mitral valve prolapse<br>Anemia<br>Autism<br>Auto-immune/rheumatological conditions<br>Cancer (all types)<br>Chronic kidney disease<br>Diabetes Type 1<br>Diabetes Type 2<br>Ehlers-Danlos Syndrome (EDS)<br>Endometriosis<br>Fibromyalgia<br>IgA deficiency<br>Interstitial Cystitis (Bladder Pain Syndrome)

	Hepatitis (A/B/C)<br>HIV<br>Mast Cell Activation Syndrome (MCAS)<br>Myalgic Encephalomyelitis / Chronic Fatigue Syndrome (ME/CFS)<br>Obesity<br>Postural Orthostatic Tachycardia Syndrome (POTS)<br>Recurrent bacterial infections<br>Recurrent viral infections<br>Restless leg syndrome<br>TMJ (temporomandibular joint dysfunction)<br>Vertigo<br>Vision: near-sighted/far-sighted<br>Vitamin D deficiency<br>None of the above<br>Other
Did any of your pre-existing conditions change during your COVID19 symptoms?	Yes, they got worse.<br>Yes, they got better.<br>No, they stayed the same.<br>N/A (I did not have any pre-existing condition)
In the month before becoming ill if you were sick, or in the previous month if you were not, were you a regular, occasional, or never smoker?	Never<br>Occasionally<br>Regularly

Hospitalization

Were you hospitalized?	Yes<br>No<br>I visited ER/Urgent care but was not admitted/did not stay overnight at a hospital
If yes: how long were you hospitalized? [Number of days]	
Did you receive oxygen support in the hospital?	Yes, nasal cannula<br>Yes, I was intubated<br>No<br>I was not hospitalized

Treatments

Have you tried any of the following treatments for your COVID19 symptoms, if	Anti-histamines<br>H1 type Antihistamines

yes, how helpful it was?<br><br>This includes Prescription or off-the-counter Medications, or Alternative Treatments.	(Diphenhydramine, acrivastine, and cetirizine, like Benadryl, Zyrtec, Claritin)<br>H2 type Antihistamines (cimetidine,famotidine, like Pepcid)<br>Products Containing Cannabis or Cannabis-derived Compounds, Including delta-9-tetrahydrocannabinol (THC) and cannabidiol (CBD)<br>Steroids<br>(Prednisone and Dexamethasone etc)<br>Blood-thinners (<br>Apixaban (Eliquis),<br>Dabigatran (Pradaxa)<br>Dalteparin (Fragmin)<br>Edoxaban (Savaysa)<br>Enoxaparin (Lovenox)<br>Fondaparinux (Arixtra)<br>Heparin (Innohep)<br>Rivaroxaban (Xarelto)<br>Warfarin (Coumadin, Jantoven)<br>Aspirin<br>Cilostazol<br>Clopidogrel (Plavix)<br>Dipyridamole (Persantine)<br>Eptifibatide (Integrilin)<br>Prasugrel (Effient)<br>Ticagrelor (Brilinta)<br>Tirofiban (Aggrastat)<br>Vorapaxar (Zontivity)<br>Remsdesevir<br>Veklury (an antiviral medicine used to treat coronavirus disease<br>Antibiotics<br>Azithromycin<br>Malaria treatments<br>Chloroquine<br>Hydroxychloroquine<br>Anti-oxidants<br>Oxaloacetate<br>Over the counter painkillers<br>Non-NSAIDs<br>(Tylenol, Paracetamol)<br>NSAIDs (Ibuprofen,Naproxen, Adult aspirin (full dose))<br>Direct oral anticoagulants<br>Rivaroxaban (Xarelto)<br>Warfarin (Coumadin)<br>Anti-inflammatories<br>Curcumin (Tumeric)<br>Omega 3 / DHA / EPA (Fish oil)<br>Intravenous gamma globulin<br>Convalescent plasma<br>None<br>Others (specify)

Vaccination

Have you got the Covid-19 vaccine?	Yes<br>No
Type of the vaccine	Comirnaty (BNT162b2) (Pfizer, BioNTech; Fosun Pharma)<br>Moderna COVID- 19 Vaccine (mRNA-1273); also called Spikevax (Moderna, BARDA, NIAID)<br>COVID-19 Vaccine AstraZeneca (AZD1222); also known as Vaxzevria and Covishield (BARDA, OWS)<br>Sputnik V (Gamaleya Research Institute, Acellena Contract Drug Research and Development, Russia)<br>Sputnik Light (Gamaleya Research Institute, Acellena Contract Drug Research and Development, Russia)<br>COVID-19 Vaccine Janssen (JNJ-78436735; Ad26.COV2.S) (Janssen Vaccines (Johnson & Johnson)<br>CoronaVac (Sinovac)<br>BBIBP-CorV (Beijing Institute of Biological Products; China National Pharmaceutical Group (Sinopharm))<br>EpiVacCorona (Federal Budgetary Research Institution State Research Center of Virology and Biotechnology)<br>Convidicea (PakVac, Ad5-nCoV) (CanSino Biologics)
How many shots have you got?	
Time of the vaccine?	Specific date
Have you got infected with COVID-19 after vaccination?	Yes<br>No

DePaul Symptom Questionnaire – 54 items

For the following questions (13-66), we would like to know **how often you have had each symptom** and **how much each symptom has bothered you over the last 6 months**. For each symptom please circle **one number for frequency and one number for severity**. Please fill the chart out from left to right.

**Symptoms**	*Frequency:*<br>Throughout the **past 6 months**, **how often** have you had this symptom?<br><br>For each symptom listed below, circle a number from:<br>**0 = none of the time**<br>**1 = a little of the time**<br>**2 = about half the time**<br>**3 = most of the time**<br>**4 = all of the time**					*Severity:*<br>Throughout the **past 6 months**, **how much** has this symptom bothered you?<br>For each symptom listed below, circle a number from:<br>**0 = symptom not present**<br>**1 = mild**<br>**2 = moderate**<br>**3 = severe**<br>**4 = very severe**				
**13) Fatigue/extreme tiredness**	0	1	2	3	4	0	1	2	3	4
**14) Dead, heavy feeling after starting to exercise**	0	1	2	3	4	0	1	2	3	4
**15) Next day soreness or fatigue after non-strenuous, everyday activities**	0	1	2	3	4	0	1	2	3	4
**16) Mentally tired after the slightest effort**	0	1	2	3	4	0	1	2	3	4
**17) Minimum exercise makes you physically tired**	0	1	2	3	4	0	1	2	3	4
**18) Physically drained or sick after mild activity**	0	1	2	3	4	0	1	2	3	4
**19) Feeling unrefreshed after you wake up in the morning**	0	1	2	3	4	0	1	2	3	4
**20) Need to nap daily**	0	1	2	3	4	0	1	2	3	4
**21) Problems falling asleep**	0	1	2	3	4	0	1	2	3	4
**22) Problems staying asleep**	0	1	2	3	4	0	1	2	3	4
**23) Waking up early in the morning (e.g. 3am)**	0	1	2	3	4	0	1	2	3	4
**24) Sleep all day and stay awake all night**	0	1	2	3	4	0	1	2	3	4
**25) Pain or aching in your muscles**	0	1	2	3	4	0	1	2	3	4
**26) Pain/stiffness/tenderness in more than one joint without swelling or redness**	0	1	2	3	4	0	1	2	3	4
**27) Eye pain**	0	1	2	3	4	0	1	2	3	4

Symptoms	*Frequency:* Throughout the **past 6 months**, **how often** have you had this symptom? For each symptom listed below, circle a number from: **0 = none of the time** **1 = a little of the time** **2 = about half the time** **3 = most of the time** **4 = all of the time**					*Severity:* Throughout the **past 6 months**, **how much** has this symptom bothered you? For each symptom listed below, circle a number from: **0 = symptom not present** **1 = mild** **2 = moderate** **3= severe** **4 = very severe**				
**28) Chest pain**	0	1	2	3	4	0	1	2	3	4
**29) Bloating**	0	1	2	3	4	0	1	2	3	4
**30) Abdomen/stomach pain**	0	1	2	3	4	0	1	2	3	4
**31) Headaches**	0	1	2	3	4	0	1	2	3	4
**32) Muscle twitches**	0	1	2	3	4	0	1	2	3	4
**33) Muscle weakness**	0	1	2	3	4	0	1	2	3	4
**34) Sensitivity to noise**	0	1	2	3	4	0	1	2	3	4
**35) Sensitivity to bright lights**	0	1	2	3	4	0	1	2	3	4
**36) Problems remembering things**	0	1	2	3	4	0	1	2	3	4
**37) Difficulty paying attention for a long period of time**	0	1	2	3	4	0	1	2	3	4
**38) Difficulty finding the right word to say or expressing thoughts**	0	1	2	3	4	0	1	2	3	4
**39) Difficulty understanding things**	0	1	2	3	4	0	1	2	3	4
**40) Only able to focus on one thing at a time**	0	1	2	3	4	0	1	2	3	4
**41) Unable to focus vision and/or attention**	0	1	2	3	4	0	1	2	3	4
**42) Loss of depth perception**	0	1	2	3	4	0	1	2	3	4
**43) Slowness of thought**	0	1	2	3	4	0	1	2	3	4
**44) Absent-mindedness or forgetfulness**	0	1	2	3	4	0	1	2	3	4
**45) Bladder problems**	0	1	2	3	4	0	1	2	3	4
**46) Irritable bowel problems**	0	1	2	3	4	0	1	2	3	4

**Symptoms**	*Frequency:*<br>Throughout the **past 6 months**, **how often** have you had this symptom?<br><br>For each symptom listed below, circle a number from:<br>**0 = none of the time**<br>**1 = a little of the time**<br>**2 = about half the time**<br>**3 = most of the time**<br>**4 = all of the time**					*Severity:*<br>Throughout the **past 6 months**, **how much** has this symptom bothered you?<br>For each symptom listed below, circle a number from:<br>**0 = symptom not present**<br>**1 = mild**<br>**2 = moderate**<br>**3= severe**<br>**4 = very severe**				
**47) Nausea**	0	1	2	3	4	0	1	2	3	4
**48) Feeling unsteady on your feet, like you might fall**	0	1	2	3	4	0	1	2	3	4
**49) Shortness of breath or trouble catching your breath**	0	1	2	3	4	0	1	2	3	4
**50) Dizziness or fainting**	0	1	2	3	4	0	1	2	3	4
**51) Irregular heart beats**	0	1	2	3	4	0	1	2	3	4
**52) Losing or gaining weight without trying**	0	1	2	3	4	0	1	2	3	4
**53) No appetite**	0	1	2	3	4	0	1	2	3	4
**54) Sweating hands**	0	1	2	3	4	0	1	2	3	4
**55) Night sweats**	0	1	2	3	4	0	1	2	3	4
**56) Cold limbs (e.g. arms, legs, hands)**	0	1	2	3	4	0	1	2	3	4
**57) Feeling chills or shivers**	0	1	2	3	4	0	1	2	3	4
**58) Feeling hot or cold for no reason**	0	1	2	3	4	0	1	2	3	4
**59) Feeling like you have a high temperature**	0	1	2	3	4	0	1	2	3	4
**60) Feeling like you have a low temperature**	0	1	2	3	4	0	1	2	3	4
**61) Alcohol intolerance**	0	1	2	3	4	0	1	2	3	4
**62) Sore throat**	0	1	2	3	4	0	1	2	3	4
**63) Tender/sore lymph nodes**	0	1	2	3	4	0	1	2	3	4
**64) Fever**	0	1	2	3	4	0	1	2	3	4
**65) Flu-like symptoms**	0	1	2	3	4	0	1	2	3	4
**66) Some smells, foods, medications, or chemicals make you feel sick**	0	1	2	3	4	0	1	2	3	4

67. Have you **always** had persistent or recurring **fatigue/energy problems**, even back to your earliest memories as a child? (By persistent or recurring, we mean that the fatigue/energy problems are usually ongoing and constant, but sometimes there are good periods and bad periods.)

 Yes No Not having a problem with fatigue/energy

68. Since your **fatigue/energy related illness** began, do your headaches either happen more often, feel worse or more severe, or are they in a different place or spot?

 Yes No Not having a problem with fatigue/energy

69. How long ago did your problem with **fatigue/energy** begin?

 Less than 6 months

 6-12 months

 1-2 years

 Longer than 2 years

 Had problem with fatigue/energy since childhood or adolescence

 Not having a problem with fatigue/energy

70. Have you been diagnosed with Chronic Fatigue Syndrome or Myalgic Encephalomyelitis?

 Yes No

70a. If yes, what year were you diagnosed? ___________________

70b. Do you currently have a diagnosis of Chronic Fatigue Syndrome or Myalgic Encephalomyelitis?
 Yes No

70c. Who diagnosed you with Chronic Fatigue Syndrome or Myalgic Encephalomyelitis?

 Medical Doctor Alternative Practitioner Self-Diagnosed

70d. Have any of your family members been diagnosed with Chronic Fatigue Syndrome or Myalgic Encephalomyelitis?
 Yes No

If yes, please list their relation to you and current age _______________________________

71. Did you experience any of the following symptoms regularly and repeatedly in the months and years <u>before</u> your fatigue/energy problems began?

- ☐ Sore throat
- ☐ Tender/sore lymph nodes
- ☐ Unrefreshing sleep
- ☐ Impaired memory and concentration
- ☐ Prolonged fatigue following physical or mental exertion
- ☐ Muscle pain
- ☐ Headaches
- ☐ Joint Pain
- ☐ Not having a problem with fatigue/energy

72. If you rest, does your problem with **fatigue/energy** go away? **(Check one)**

☐ Entirely

☐ Partially

☐ My fatigue/energy problem is not improved by rest *(Skip to Question 73)*

☐ I am not having a problem with fatigue/energy *(Skip to Question 73)*

72a. How long do you have to rest for your problem with **fatigue/energy** to entirely or partially go away?
☐ less than 30 minutes ☐ 30 to 59 minutes ☐ 1-2 hours ☐ more than 2 hours

73. If you were to become exhausted after actively participating in extracurricular activities, sports, or outings with friends, would you recover within an hour or two after the activity ended?

☐ Yes ☐ No

74. Do you reduce your activity level to avoid experiencing problems with **fatigue/energy**?

☐ Yes ☐ No ☐ Not having a problem with fatigue/energy

75. Do you experience a worsening of your **fatigue/energy related illness** after engaging in minimal physical effort?

☐Yes ☐ No ☐ Not having a problem with fatigue/energy

75a. Do you experience a worsening of your **fatigue/energy related illness** after engaging in mental effort?
□ Yes □ No

75b. If you feel worse after activities, how long does this last? **(Check one)**
□ 1 hour or less -3□ Hrs 2 -10□ 4 Hrs -13□

11 Hrs □ 14-23 Hrs (□Please specify__________) More than

24 Hrs

76. Are you currently engaging in any form of exercise?

□ Yes *(Skip to Question 77)* □ No

76a. If you do not exercise, why aren't you exercising? **(Check all boxes that you agree with)**

□ Not interested

□ No time

□ Would like to but cannot because of problems with fatigue/energy □

Cannot because exercise makes symptoms worse

77. Over what period of time did your **fatigue/energy related illness,** develop? **(Check one)**

□ Within 24 hours

□ Over 1 week

□ Over 1 month

□ Over 2-6 months

□ Over 7-12 months

□ Over 1-2 years

□ Longer than 2 years

□ Had problem with fatigue/energy since childhood or adolescence

□ I am not ill

78. How would you describe the course of your **fatigue/energy related illness? (Check one)**

- Constantly getting worse
- Constantly improving
- Persisting (no change)
- Relapsing & remitting (having "good" periods with no symptoms & "bad" periods)
- Fluctuating (symptoms periodically get better and get worse, but never disappear completely)
- No Symptoms/I am not ill

79. Which statement best describes your **fatigue/energy related illness** during the **<u>last 6 months</u>**? **(Check one)**

- I am not able to work or do anything, and I am bedridden.
- I can walk around the house, but I cannot do light housework.
- I can do light housework, but I cannot work part-time.
- I can only work part-time at work or on some family responsibilities.
- I can work full time, but I have no energy left for anything else.
- I can work full time and finish some family responsibilities but I have no energy left for anything else.
- I can do all work or family responsibilities without any problems with my energy.

80. Did your **fatigue/energy related illness** start after you experienced any of the following?
(Check one or more and please specify)

- An infectious illness____
- An accident____
- A trip or vacation____
- An immunization (shot at doctor's office)____________________________
- Surgery____
- Severe stress (bad or unhappy event(s))_____________________________
- Other____
- I am not ill

81. Have you ever consulted a medical doctor or health professional about your **fatigue/energy** problem?

 Yes No *(Skip to Question 83)*

82. Do you currently have a medical doctor overseeing your **fatigue/energy** problem?

☐ Yes ☐ No

83. Do you have any medical illness (es) that might be causing your symptoms?

☐ Yes ☐ No *(Skip to Question 84)*

83a. What medical illnesses do you have?

Illness name(s) and year it began: ____________________

____

____

____

83b. For which of these conditions are you currently receiving treatment? ____________________

____

____

____

84. Are you currently taking any medications (over the counter or prescription)?

☐ Yes ☐ No *(Skip to Question 86)*

84a. What medications are you taking? ____________________

____

85. Do you think any medication(s) is (are) causing your problem with **fatigue/energy**?

☐ Yes ☐ No *(Skip to Question 86)*

☐ I do not have a problem with fatigue/energy *(Skip to Question 86)*

85a. Please specify which medications: ____________________

____

86. Have you ever been diagnosed and/or treated for any of the following: **(Check all that apply and write year (s) experienced, years treated, and medication (if applicable) in the blank)**

 Major depression____
 Major depression with melancholic or psychotic features__________________
 Bipolar disorder (Manic-depression)___________________________
 Anxiety____
 Schizophrenia____
 Eating disorder____
 Substance abuse____
 Multiple chemical sensitivities_____________________________
_____ Fibromyalgia_____________________________________
____
 Allergies____
 Other (*Please specify*)______________________________________

 No diagnosis/treatment

87. What do you think is the cause of your problem with **fatigue/energy**? **(Check one)**

 Definitely physical

 Mainly physical

 Equally physical and psychological

 Mainly psychological

 Definitely psychological

 No problem with fatigue/energy

88. Do you think anything specific in your personal life or environment accounts for your problem with **fatigue/energy**?

 Yes No *(Skip to Question 89)*

 I do not have a problem with fatigue/energy (*Skip to Question 89)*

88a. Please specify:____

89. In the **past 4 weeks**, approximately how many hours per week have you spent doing:

Household related activities?____________hours per week

Social/Recreational related activities?_____hours per week

Family related activities?_______________hours per week

Work related activities?________________hours per week

90. In the **past 4 weeks**, have you had to reduce the number of hours you previously spent (prior to your illness) on occupational, social or family activities because of your health or problems with **fatigue/energy**?

 Yes No *(Skip to Question 91)* Not having a problem with fatigue/energy 90a. **Before your fatigue/energy related illness**, approximately how many hours did you used to spend on:

Household related activities? ____________hours per week

Social/Recreational related activities? _____hours per week

Family related activities? _______________hours per week

Work related activities?_________________hours per week

NOTE: For those people who are NOT having a problem with fatigue/energy, please answer questions 91-96 assuming that a score of 100= having abundant energy that allows one to work full-time and perform daily chores.

91. Please rate the amount of **energy** you had available **yesterday,** using a scale from 1 to 100 where 1 = no energy and 100 = your pre-illness energy level_____________ __

92. Please rate the amount of **energy** you expended (used) **yesterday,** using a scale from 1 to 100 where 1 = no energy and 100 = your pre-illness energy expended______________

93. Please rate the amount of **fatigue** you had **yesterday,** using a scale from 1 to 100 where 1 = no fatigue and 100 = severe fatigue______________

94. For the **past week,** please rate the amount of **energy** you had available using a scale from 1 to 100 where 1=no energy and 100=your pre-illness energy level_____________

95. For the **past week,** please rate the amount of **energy** you have expended (used) using a scale from 1 to 100 where 1 = no energy and 100 = your pre-illness energy expended_ ________

96. For the **past week,** please rate the amount of **fatigue** you have had using a scale from 1 to

100 where 1 = no fatigue and 100 = severe fatigue ________________

MOS SURVEY

INSTRUCTIONS:

This survey asks for your views about your health. This information will help keep track of how you feel and how well you are able to do your usual activities. Answer every question by marking the answer as indicated. If you are unsure about how to answer a question, please give the best answer you can.

1. In general, would you say your health is: ***(Please circle one)*** Excellent .... 1 Very good .... 2 Good .... 3 Fair 4
Poor 5

2. **Compared to one year ago,** how would you rate your health in general now? ***(Please circle one)***

Much better than one year ago 1
Somewhat better now than one year ago 2
About the same as one year ago 3
Somewhat worse now than one year ago 4
Much worse now than one year ago 5

3. The following items are about activities you might do during a typical day. Does your health now limit you in these activities? If so, how much?

**Activities**	**Yes, Limited A Lot**	**Yes, Limited A Little**	**No, Not Limited At All**
**Vigorous activities**: running, lifting heavy objects, participating in strenuous sports	1	2	3
**Moderate activities**: moving a table, pushing a vacuum cleaner, bowling, playing golf	1	2	3
Lifting or carrying groceries	1	2	3
Climbing **several** flights of stairs	1	2	3
Climbing **one** flight of stairs	1	2	3
Bending, kneeling, or stooping	1	2	3
Walking **more than a mile**	1	2	3
Walking **several blocks**	1	2	3
Walking **one** block	1	2	3
Bathing or dressing yourself	1	2	3

4. During the **past 4 weeks**, have you had any of the following problems with your work or other regular daily activities as a result of your **physical health**?

**Problems**	**Yes**	**No**

Cut down on the **amount of time** you spent on work or other activities	1	2
**Accomplished less** than you would like	1	2
Were limited in the **kind** of work or other activities	1	2
Had **difficulty** performing the work or other activities (For example, it took extra effort)	1	2

5. During the **past 4 weeks**, have you had any of the following problems with your work or other regular daily activities **as a result of any emotional problems** (such as feeling depressed or anxious)?

**Problems**	**Yes**	**No**
Cut down the **amount of time** you spent on work or other activities	1	2
**Accomplished less** than you would like	1	2
Didn't do work or other activities as **carefully** as usual	1	2

6. During the **past 4 weeks**, to what extent has your physical health or emotional problems interfered with your normal social activities with family, neighbors, or groups? ***(Please circle one)***

Not at all .... 1 Slightly
.... 2 Moderately
.... 3 Quite a bit
.... 4
Extremely .... 5 7. How much

bodily pain have you had during the **past 4 weeks**?

None .... 1 Very mild
.... 2 Mild
.... 3 Moderate
.... 4 Severe
.... 5 Very Severe
.... 6

8. During the **past 4 weeks**, how much did pain interfere with your normal work (including both work outside the home and housework)?

Not at all 1
Slightly 2
Moderately 3
Quite a bit 4
Extremely 5

9. These questions are about how you feel and how things have been with you **during the past 4 weeks**.

For each question, please give the one answer that comes closest to the way you have been

feeling. How much of the time **during the past 4 weeks**-

**Questions**	**All of the Time**	**Most of the Time**	**A Good Bit of the Time**	**Some of the Time**	**A Little of the Time**	**None of the Time**
Did you feel full of pep?	1	2	3	4	5	6
Have you been a nervous person?	1	2	3	4	5	6
Have you felt so down in the dumps that nothing could cheer you up?	1	2	3	4	5	6
Have you felt calm and peaceful?	1	2	3	4	5	6
Did you have a lot of energy?	1	2	3	4	5	6
Have you felt down-hearted and blue?	1	2	3	4	5	6
Did you feel worn out?	1	2	3	4	5	6
Have you been a happy person?	1	2	3	4	5	6
Did you feel tired?	1	2	3	4	5	6

10. During the **past 4 weeks**, how much of the time has your physical health or emotional problems interfered with your social activities (like visiting with friends, relatives, etc.)?

All of the time 1
Most of the time 2
Some of the time 3
A little of the time 4
None of the time 5

11. How **TRUE** or **FALSE** is each of following statements for you?

**Statements**	**Definitely True**	**Mostly True**	**Don't Know**	**Mostly False**	**Definitely False**
I seem to get sick a little easier than other people	1	2	3	4	5
I am as healthy as anybody I know	1	2	3	4	5
I expect my health to get worse	1	2	3	4	5
My health is excellent	1	2	3	4	5

Patient Health Questionnaire (PHQ-9)

Over the last two weeks, how often have you been bothered by any of the following problems?	Not at all	Several days	More than half the days	Nearly every day
Little interest or pleasure in doing things?				

Feeling down, depressed, or hopeless?				
Trouble falling or staying asleep, or sleeping too much?				
Feeling tired or having little energy?				
Poor appetite or overeating?				
Feeling bad about yourself - or that you are a failure or have let yourself or your family down?				
Trouble concentrating on things, such as reading the newspaper or watching television?				
Moving or speaking so slowly that other people could have noticed? Or the opposite - being so fidgety or restless that you have been moving around a lot more than usual?				
Thoughts that you would be better off dead, or of hurting yourself in some way?				

Other symptoms

1. Were you given any of these diagnoses for any of your symptoms?<br>Was this:<br>Before COVID-19 infection<br>After COVID-19 infection<br>After COVID-19 vaccine<br>Unrelated to COVID	Guillain-Barre Syndrome<br>Small fiber neuropathy Autonomic neuropathy<br>Polyneuropathy<br>Neuralgia (please include the type of neuralgia in the text box)<br>Antiphospholipid Syndrome, viral-induced or autoimmune<br>Sarcoidosis<br>Stroke (please include the type of stroke in the text box)<br>Demyelinating lesions<br>POTS<br>Encephalopathy<br>Encephalitis (please include the type of encephalitis in the text box)<br>Meningoencephalitis<br>Meningitis<br>Acute Disseminated Encephalomyelitis<br>Acute myelitis<br>Ophthalmo-paresis<br>Psychiatric Diagnosis<br>Migraine<br>Motor Peripheral or Cranial Neuropathies

	Posterior reversible encephalopathy syndrome<br>Myasthenia<br>Thrombotic microangiopathy<br>Tapia Syndrome<br>Epilepsy<br>Traumatic Brain Injury (TBI) or TBI-like symptoms<br>Myalgic Encephalomyelitis / Chronic Fatigue Syndrome (ME/CFS)<br>Cranial nerve involvement<br>Macular hole<br>Costochondritis<br>Blood clot<br>Myocarditis
Memory Symptoms<br>2. Have you experienced any MEMORY RELATED SYMPTOMS since the start of your COVID-19 illness? *	Yes<br>No
3. Which of the following memory symptoms have you experienced since the start of your COVID-19 illness? *<br>Was this:<br>Before COVID-19 infection<br>After COVID-19 infection<br>After COVID-19 vaccine<br>Unrelated to COVID	Short-term memory loss (memory that lasts ~30 seconds, i.e.remembering a phone number before writing it down, or forgetting you're in the middle of a task)<br>Long-term memory loss (long-term memory can be anything from remembering yesterday, forgetting you've done a task, forgetting recently learned information, or forgetting your third-grade experience)<br>Not being able to make new memories<br>Forgetting how to do routine tasks (tying your shoelaces, washing your hands) None of the above<br>Other
Cognitive Function/Brain Fog Symptoms<br>4. Have you experienced issues with BRAIN FOG (inability to focus, think clearly, plan, process, understand, and maintain a coherent stream of thought; abnormally slow or fast thoughts) since the start of your COVID-19 illness? *	Yes<br>No
5. Which of the following brain fog/cognitive functioning symptoms have you experienced since the start of your COVID-19 illness? *<br>Was this:<br>Before COVID-19 infection	Difficulty with executive functioning (planning, organizing, figuring out the sequence of actions, abstracting)<br>Agnosia (failure to recognize or identify objects despite intact sensory functioning)

After COVID-19 infection<br>After COVID-19 vaccine<br>Unrelated to COVID	Difficulty problem-solving or decision-making<br>Difficulty thinking<br>Thoughts moving too quickly<br>Slowed thoughts<br>Poor attention or concentration<br>I did NOT have any Brain Fog symptoms<br>Other
Emotional/Behavioral Changes Emotional and Behavioral Changes<br>6. Compared to how you felt before COVID, have you experienced an increase in any of the following? *	Difficulty controlling your emotions<br>Lack of inhibition (difficulty controlling your behavior)<br>Irritability<br>Anger<br>Impulsivity (acting on a whim without self-control)<br>Aggression<br>Euphoria (a feeling or state of intense excitement and happiness)<br>Delusions<br>Depression<br>Apathy (lack of feeling, emotion, interest, or concern)<br>Suicidality<br>Mood swings<br>Anxiety<br>Mania (abnormally elevated/excited mood, decreased need for sleep, occasionally with delusions) Hypomania (a milder form of mania)<br>Tearfulness<br>Sense of doom<br>None of the above<br>Other
Speech and Other Language Issues<br>7. Have you experienced any issues with SPEECH AND LANGUAGE since the start of your COVID-19 illness? *	Yes<br>No
8. Which of the following speech and language symptoms have you experienced since the start of your COVID-19 illness? *<br>Was this:<br>Before COVID-19 infection<br>After COVID-19 infection<br>After COVID-19 vaccine<br>Unrelated to COVID	Difficulty finding the right words while speaking/writing<br>Difficulty communicating verbally<br>Difficulty speaking in complete sentences<br>Speaking unrecognizable words<br>Difficulty communicating in writing<br>Difficulty processing/understanding what others say<br>Difficulty reading/processing written text<br>(If applicable) changes to your non-primary

	(second/third) language skills<br>Other
Headaches<br>9. Have you experienced any new HEADACHES OR RELATED ISSUES since the start of your COVID-19 illness? *	Yes<br>No
10. Which of the following symptoms have you experienced since the start of your COVID-19 illness? *<br>Was this:<br>Before COVID-19 infection<br>After COVID-19 infection<br>After COVID-19 vaccine<br>Unrelated to COVID	Headaches, at the base of the skull<br>Headaches, in the temples<br>Headaches, behind the eyes<br>Headaches, diffuse (entire brain)<br>Headaches/pain after mental exertion<br>Sensation of brain warmth/"on fire"<br>Sensation of brain pressure<br>Migraines<br>None of the above
Sense of Smell and Taste<br>11. Have you experienced any changes to your SENSE OF SMELL OR TASTE since the start of your COVID-19 illness? *	Yes<br>No
12. Which of the following symptoms have you experienced since the start of your COVID-19 illness? *<br>Was this:<br>Before COVID-19 infection<br>After COVID-19 infection<br>After COVID-19 vaccine<br>Unrelated to COVID	Loss of smell<br>Phantom smells (imagining/hallucinating smells - smelling things that aren't there)<br>Heightened sense of smell<br>Altered sense of smell<br>Loss of taste<br>Phantom taste (imagining/hallucinating tastes - tasting things when there's nothing in your mouth)<br>Heightened sense of taste<br>Altered sense of taste<br>None of the above
Tremors and Vibrating Sensations<br>13. Have you experienced any TREMOR OR VIBRATION SENTATIONS since the start of your COVID-19 illness? *<br>Tremor: Involuntary, rhythmic muscle contraction leading to shaking movements in one or more parts of the body<br>Vibration sensation: A buzzing feeling, when you feel like your muscles, fingers, or legs are vibrating or shaking inside, but you don't see the movement	Yes<br>No
Sleeping issues	Yes

14. Have you experienced any SLEEPING ISSUES since the start of your COVID-19 illness? *	No
15. Which of the following sleeping issues have you experienced since the start of your COVID-19 illness? *<br>Was this:<br>Before COVID-19 infection<br>After COVID-19 infection<br>After COVID-19 vaccine<br>Unrelated to COVID	Lucid dreams (dreams where you are aware you are dreaming or have some control over what you dream about)<br>Vivid dreams<br>Nightmares<br>Insomnia<br>Night sweats<br>Restless leg syndrome<br>Awakened by feeling like you couldn't breathe<br>Sleep apnea<br>Other
16. If you have/had insomnia, which best describes the type of insomnia? *	Difficulty falling asleep<br>Waking up early in the morning<br>Waking up several times during the night<br>None of the above
17. What is causing/caused your insomnia? *	Pain<br>Sensitivity to outside light/noise<br>Other physical discomfort<br>Anxiety/depression/racing thoughts<br>Difficulty breathing<br>A sensation of adrenaline/energy<br>A sensation like the virus was keeping me awake<br>Other
Hallucinations<br>18. Have you experienced any HALLUCINATIONS (visual, hearing, or touch) since the start of your COVID-19 illness? *	Yes<br>No
19. Which of the following hallucinations have you experienced since the start of your COVID-19 illness? *<br>Was this:<br>Before COVID-19 infection<br>After COVID-19 infection<br>After COVID-19 vaccine<br>Unrelated to COVID	Visual (seeing) Hallucinations<br>Auditory (hearing) Hallucinations<br>Tactile (touch) Hallucinations<br>Hallucinations<br>other
20. Which of the following NEUROLOGICAL SENSATION SYMPTOMS have you experienced since the start of your COVID-19 illness, if any? *<br>Please specify the location on your body in the text box. If multiple locations, please separate them with a comma (i.e. hand, leg, foot).	Skin sensations: burning, tingling, or itchiness without rash Numbness/loss of sensation<br>Numbness/weakness on one side of the body only<br>Coldness<br>Tingling/prickling/pins and needles sensation<br>Electrical zaps/electrical shock sensation<br>Facial paralysis (please indicate where on face was paralyzed)

	Sensation of facial pressure/numbness, left side<br>Sensation of facial pressure/numbness, right side<br>Sensation of facial pressure/numbness, other:<br>None of the above
Temperature Issues<br>21. Have you experienced any TEMPERATURE ISSUES (including heat intolerance, chills, high/low temperature) since the start of your COVID-19 illness? *	Yes<br>No
22. Did you experience any of the following TEMPERATURE ISSUES since the start of your COVID-19 illness?<br>Was this:<br>Before COVID-19 infection<br>After COVID-19 infection<br>After COVID-19 vaccine<br>Unrelated to COVID	Temperature lability (quick swings in and out of fever or elevated temperature)<br>Heat intolerance<br>Other temperature issues (not listed above or below)
Cardiovascular Symptoms<br>23. Which of the following symptoms have you experienced? *<br>Was this:<br>Before COVID-19 infection<br>After COVID-19 infection<br>After COVID-19 vaccine<br>Unrelated to COVID	Tachycardia<br>(high heart rate, >90 beats per minute)<br>Bradycardia(low heart rate, <60 beats per minute)<br>Heart palpitations (sensation or awareness of your heart beating. Feeling like your heart is racing, thumping or skipping beats)<br>Abnormally high blood pressure<br>Abnormally low blood pressure<br>Visibly inflamed/bulging veins<br>Fainting<br>Blood clots(Thrombosis)
24. Generic Issues<br>Was this:<br>Before COVID-19 infection<br>After COVID-19 infection<br>After COVID-19 vaccine<br>Unrelated to COVID	Dizziness / vertigo /unsteadiness or balance issues<br>Neuralgia (nerve pain)<br>Seizures (confirmed)<br>Seizures (suspected)<br>Episodes of breathing difficulty/gasping for air when your oxygen saturation is normal<br>Low oxygen levels (<94%)<br>New/unexpected anaphylaxis reaction<br>Acute (sudden) confusion/disorientation<br>Slurring words/speech<br>High blood sugar (if measured)<br>Low blood sugar (if measured)
Gastrointestinal Issues<br>25. Have you experienced any Gastrointestinal Issues since the start of your COVID-19 illness? *<br>26. Which of the following Gastrointestinal Issues have you experienced since the start of your COVID-19 illness? *	None of the below gastrointestinal symptoms apply to me<br>Constipation<br>Diarrhea<br>Vomiting<br>Nausea

Was this:<br>Before COVID-19 infection<br>After COVID-19 infection<br>After COVID-19 vaccine<br>Unrelated to COVID	Loss of Appetite<br>Abdominal pain<br>Lower<br>Esophagus<br>Burning /<br><br>gastroesophageal reflux / acid reflux
Respiratory and Sinus Symptoms<br>27. Have you experienced any Respiratory and Sinus Symptoms since the start of your COVID-19 illness? *<br>28. Which of the following Respiratory and Sinus Symptoms have you experienced since the start of your COVID-19 illness? *	None of the below respiratory and sinus symptoms apply to me<br>Dry cough<br>Cough with mucus production<br>Coughing up Blood<br>Shortness of Breath<br>Tightness of Chest<br>Sneezing<br>Runny nose<br>Pain/burning in chest<br>Rattling of breath<br>Sore Throat
Skin and Allergy Symptoms<br>29. Have you experienced any Skin and Allergy Symptoms since the start of your COVID-19 illness? *<br>30. Which of the following Skin and Allergy Symptoms have you experienced since the start of your COVID-19 illness?<br>Was this:<br>Before COVID-19 infection<br>After COVID-19 infection<br>After COVID-19 vaccine<br>Unrelated to COVID	None of the below skin and allergy symptoms apply to me<br>Skin and Allergy Symptoms<br>Peeling skin<br>Petechiae (tiny purple, red, or brown spots on the skin, usually on arms, legs, stomach, buttocks, and occasionally inside mouth or on eyelids)<br>COVID toes (discoloration, swelling, painful, or blistering toes)<br>Dermatographia (writing on your<br><br>skin causes red lines where you scratched)<br>New allergies (food, chemical, environmental, etc)<br>Skin rashes<br>Other
Muscle and Joint issues<br>31. Have you experienced any Muscle and Joint issues since the start of your COVID-19 illness? *<br><br>32. Which of the following Muscle and Joint issues have you experienced since the start of your COVID-19 illness?<br>Was this:<br>Before COVID-19 infection<br>After COVID-19 infection<br>After COVID-19 vaccine<br>Unrelated to COVID	Muscle and Joint issues<br>Muscle spasms<br>Muscle aches<br>Joint pain<br>Bone ache or burning<br>None of the above

All Other Symptoms<br>33. Have you experienced any of these symptoms since the start of your COVID-19 illness? *<br>Was this:<br>Before COVID-19 infection<br>After COVID-19 infection<br>After COVID-19 vaccine<br>Unrelated to COVID	(Please choose all options that apply)<br>Inability to cry<br>Inability to yawn<br>Lump in throat/difficulty swallowing<br>Changes in the voice<br>Coughing up Blood<br>Feeling like you aren't real/like you're observing yourself from outside your body (depersonalization)<br>Feeling like the world isn't real (derealization)<br>Extreme thirst<br>None of the above
Ear and Hearing<br>34. Have you experienced any Ear and Hearing since the start of your COVID-19 illness? *<br>35. Which of the following Ear and Hearing have you experienced since the start of your COVID-19 illness?<br>Was this:<br>Before COVID-19 infection<br>After COVID-19 infection<br>After COVID-19 vaccine<br>Unrelated to COVID	Ear pain<br>Changes to the ear canal (such as pressure, blockage, burning, swelling)<br>Numbness/loss of sensation<br>Sensitivity to noise<br>Other ear/hearing symptoms<br>None of the above
Eye and Vision<br>36. Have you experienced any Eye and Vision since the start of your COVID-19 illness? *<br>37. Which of the following Eye and Vision have you experienced since the start of your COVID-19 illness?<br>Was this:<br>Before COVID-19 infection<br>After COVID-19 infection<br>After COVID-19 vaccine<br>Unrelated to COVID	Vision symptoms - Blurred vision<br>Vision symptoms - Double vision<br>Vision symptoms - Sensitivity to light<br>Vision symptoms - Tunnel vision<br>Vision symptoms - Total loss of vision<br>Eye pressure or pain<br>Pink eye (conjunctivitis)<br>Bloodshot eyes<br>Dry eyes<br>Redness on the outside of eyes<br>Floaters<br>Seeing things in your peripheral vision<br>Other eye issues:<br>None of the above
Reproductive and Urinary Symptoms<br>38. Have you experienced any Reproductive and Urinary Symptoms since the start of your COVID-19 illness? *<br>39. Which of the following Reproductive and Urinary Symptoms have you experienced since the start of your COVID-19 illness? Urinary<br>Was this:<br>Before COVID-19 infection<br>After COVID-19 infection<br>After COVID-19 vaccine	Early Menopause<br>Post-Menopausal bleeding/spotting<br>Abnormally heavy periods/clotting<br>Abnormally irregular periods<br>Other menstrual issues<br>Decrease in size of testicles/penis<br>Pain in testicles<br>Other semen/penis/testicles issues<br>Sexual dysfunction (difficulty maintaining erection, vaginal dryness,<br>Bladder control

Unrelated to COVID	
Gastrointestinal issues<br>40. Have you experienced any Gastrointestinal issues since the start of your COVID-19 illness? *<br>41. Which of the following Gastrointestinal issues have you experienced since the start of your COVID-19 illness? Urinary<br>Was this:<br>Before COVID-19 infection<br>After COVID-19 infection<br>After COVID-19 vaccine<br>Unrelated to COVID	Feeling full quickly when eating<br>Abdominal pain<br>Hyperactive bowel sensations<br>None of the above
Skin and Allergy<br>42. Have you experienced any Skin and Allergy since the start of your COVID-19 illness? *<br>43. Which of the following Skin and Allergy have you experienced since the start of your COVID-19 illness? Urinary<br>Was this:<br>Before COVID-19 infection<br>After COVID-19 infection<br>After COVID-19 vaccine<br>Unrelated to COVID	New allergies (food, chemical, environmental, etc)<br>Heightened reaction to old allergies<br>Itchy skin<br>Itchy eyes<br>Itchy, other<br>Brittle/discolored nail<br>Shingles<br>None of the above
44. General Functioning<br>Was this:<br>Before COVID-19 infection<br>After COVID-19 infection<br>After COVID-19 vaccine<br>Unrelated to COVID	In general, would you say your health BEFORE the onset of COVID was: *<br>Excellent<br>Very good<br>Good<br>Fair<br>Poor
Mental Health<br>45. Have you ever (before COVID-19 symptoms) been diagnosed with a mental health condition (e.g. depression, anxiety, panic disorder, psychosis, etc.)?	Yes<br>No
46. Do you believe you have or have had a mental health condition that has not been diagnosed?	Yes<br>No
47. If you answered yes to either question above, Which of the following have you experienced? (check all that apply)<br>Was this:<br>Before COVID-19 infection<br>After COVID-19 infection<br>After COVID-19 vaccine<br>Unrelated to COVID	Depression<br>Bipolar Disorder<br>Anxiety Disorder<br>Substance Use Disorder<br>Eating Disorder<br>Personality Disorder<br>Psychotic Disorder<br>Delirium<br>Post-traumatic stress disorder (PTSD)<br>Other
Equilibrium disorders (vertigo/dizziness)	

48.	
49. Have you ever experienced vertigo/dizziness before COVID-19 diagnosis?	(yes/no)
50. Have you started experiencing dizziness or vertigo after diagnosis of COVID-19?	(yes/no)
51. If YES, please describe the characteristics of your symptoms<br>52. Indicate the severity of your vertigo/dizziness (0–10)	violent vertigo attacks<br>chronic dizziness<br>instability
Tinnitus<br>53. Have you ever experienced tinnitus before COVID-19 diagnosis?	(yes/no)
54. Have you started experiencing tinnitus after diagnosis of COVID-19?	(yes/no)
55. If yes, please specify the characteristics of your tinnitus Indicate the severity of your tinnitus (0–10)	occasional<br>continuous floating<br>persistent<br>pulsatile<br>continuous
Migraine<br>56. Do you suffer from migraine? (yes/no)	
